# Comparison of two intraoral scanners based on three-dimensional surface analysis

**DOI:** 10.1186/s40510-018-0205-5

**Published:** 2018-02-12

**Authors:** Kyung-Min Lee

**Affiliations:** Department of Orthodontics, School of Dentistry, Chonnam National University, 33 Yongbong-ro, Buk-gu, Gwangju, 61186 South Korea

**Keywords:** Intraoral scan, Three-dimensional surface analysis, Digital impression

## Abstract

**Background:**

This in vivo study evaluated the difference of two well-known intraoral scanners used in dentistry, namely iTero (Align Technology) and TRIOS (3Shape).

**Methods:**

Thirty-two participants underwent intraoral scans with TRIOS and iTero scanners, as well as conventional alginate impressions. The scans obtained with the two intraoral scanners were compared with each other and were also compared with the corresponding model scans by means of three-dimensional surface analysis. The average differences between the two intraoral scans on the surfaces were evaluated by color-mapping. The average differences in the three-dimensional direction between each intraoral scans and its corresponding model scan were calculated at all points on the surfaces.

**Results:**

The average differences between the two intraoral scanners were 0.057 mm at the maxilla and 0.069 mm at the mandible. Color histograms showed that local deviations between the two scanners occurred in the posterior area. As for difference in the three-dimensional direction, there was no statistically significant difference between two scanners.

**Conclusions:**

Although there were some deviations in visible inspection, there was no statistical significance between the two intraoral scanners.

## Background

With the advances in computer technology, digital dental models are now being widely used for orthodontic diagnosis and treatment planning. The use of digital models alleviates many of the challenges posed by plaster models made from conventional impressions, which include the burden of storage, the risk of damage or breakage, and the difficulties in sharing the data with other clinicians involved in the patients’ care [1, 2]. Digital dental models can be created through either indirect or direct techniques. Indirect methods involve laser scanning or computed tomographic imaging of the alginate impressions or plaster models, and direct methods involve intraoral scanners. With the introduction of chairside intraoral scanners, interest in obtaining digital dental model using the direct method has increased [3–5].

After the introduction of computer-aided design/computer-aided manufacturing (CAD/CAM) concepts into dental applications by Dr. Francois Duret at the Chicago Midwinter Meeting in 1989 [6, 7], several intraoral scanners have been introduced. Recently, a few intraoral scanners have been released on the market, including the iTero (Align Technologies), TRIOS (3Shape), True Definition (3M ESPE), CEREC Omnicam (Sirona), and CS 3600 (Carestream Dental) [8, 9].

The accuracy of intraoral scanners has been evaluated for both single abutment [10–13] and short-span fixed dental prostheses [14–16]. To determine the accuracy of intraoral scanners, researchers have performed in vitro studies using reference models [17–20]. Although short-span intraoral scans have exhibited excellent accuracy, few in vivo studies have investigated the accuracy of intraoral scans on whole dentition in the clinical setting. Kuhr et al. [21] developed a new method of measuring the trueness of full-arch intraoral scans using reference spheres. However, the authors investigated only lower teeth [21]. Anh et al. [22] compared the precision of images acquired using the iTero and TRIOS intraoral scanners; however, they performed in vitro studies using fabricated dental arch models.

The iTero and TRIOS scanners allow full-arch scanning and do not require powdering of the tooth surfaces. Moreover, these two scanners have incorporated the orthodontic application software within the scanners. To our knowledge, iTero and TRIOS are the two best-known commercially available intraoral scanners in dentistry; therefore, it is important to compare their relative scanning accuracy (Table 1). We cannot assume that all intraoral scanners will produce the same level of clinically acceptable results, and it would be beneficial to see how the measurements compare between different scanners. With this in mind, the present study aimed to compare the scanning results of the iTero and TRIOS scanners.

**Table 1:** Two commercially available intraoral scanners used in this study

Scanner	Manufacturer	Software application	Scanning technology	Light source
iTero	Align Technology	iOC	Parallel confocal microscopy	Laser
TRIOS	3Shape	OrthoAnalyzer	Confocal microscopy	Laser

## Methods

Thirty-two participants were enrolled in the study after providing informed consent. This study was approved by the Institutional Review Board for Medical Science at the Chonnam National University Hospital, Gwangju, Korea (CNUDH-2015-003). The inclusion criteria were complete permanent dentition, with no missing tooth and no crown or bridge restoration. In addition, participants with moderate or severe crowding and dentofacial deformity were excluded from this study.

### Intraoral scanning with TRIOS and iTero

Intraoral scans of 32 participants were included in the present study. Each participant underwent intraoral scanning with TRIOS (3Shape, Copenhagen, Denmark) and iTero (version 4.0; Align Technology, San Jose, CA), as well as alginate impression. All intraoral scans with the iTero and TRIOS scanners were recorded by a single examiner. The scanners were calibrated every 8 days according to the manufacturers’ recommendation. TRIOS scanning was performed following the instructions of the manufacturer. In brief, the scanning was started from the left side and continued to the right side along the occlusion. After the occlusal surfaces were scanned, lingual and buccal surface scans were performed. In the upper arch, the occlusal surfaces were scanned first, in the same manner as in the lower arch, whereas the buccal and lingual surfaces were scanned in order. When scanning the occlusal surfaces, the scanner head was kept at 0–5 mm from the tooth. For the scanning of the buccal and lingual surfaces, the scanner tip was rolled 45°–90° to the buccal and lingual sides, respectively. The image could be continuously viewed on a screen during the scanning process, which allowed direct visual feedback to ensure that no areas were missed. After scanning, all scan data were sent to the OrthoAnalyzer™ (3Shape, Copenhagen, Denmark) software program, where they were reprocessed as a stereolithography (STL) file. The iTero scanning was performed in a predetermined sequence. The mandibular left area was scanned from the second molar to the incisor. The mandibular right area was scanned from the second molar. In the maxilla, the right quadrant was scanned first. The scan data were reprocessed as an STL file.

### Conventional alginate impression taking

Alginate impressions (Cavex Normal set; Cavex Holland BV, Haarlem, The Netherlands) for the maxilla and mandible were taken in a metal tray. Each impression was rinsed with tap water and disinfected via spraying (CONTINU Dental Impression Disinfectant, Premium Plus, Bournemouth, UK). After disinfection, the impression was directly poured with dental stone. The stone casts were stored for 5–7 days at a temperature of 23 °C ± 1 °C and humidity of 40% ± 10%. The dental models were scanned with a laboratory scanner (Orapix, Seoul, Korea). By means of a reverse engineering software program (Rapidform 2006; 3D Systems, Rock Hill, SC), the laboratory-scanned file was converted to STL format. The adjacent gingival tissue was deleted along the margin of the clinical crown to allow accurate best-fit alignment of the crowns.

### Comparison of the two intraoral scanners based on three-dimensional surface analysis

**Fig. 1:**
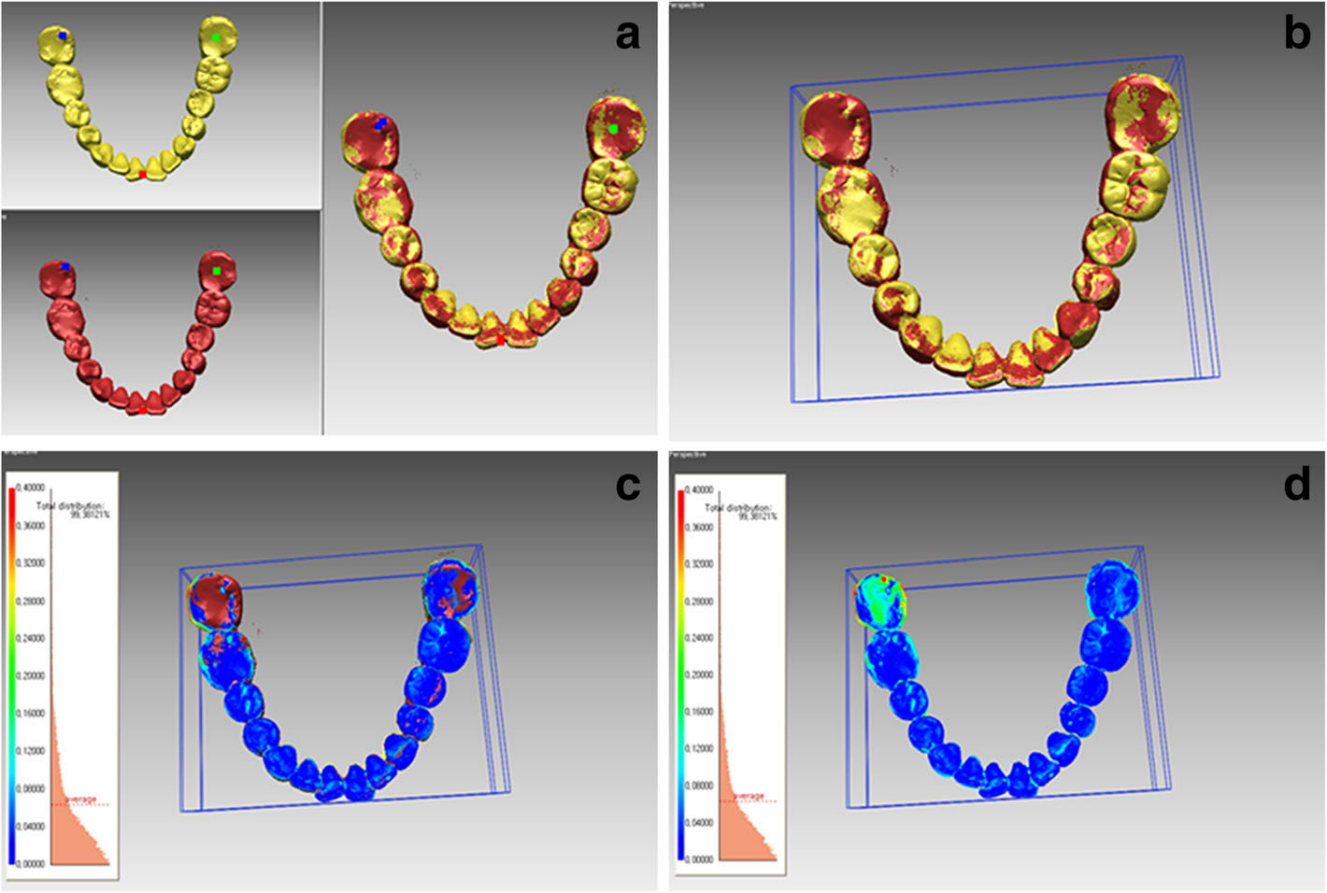
Registration of the three-dimensional models created by iTero (yellow) and TRIOS (red). **a** Initial registration by three corresponding points on each of the two models. **b** Automatic fine registration for final registration. **c** Computation of average deviations between the two intraoral scans at all points on the surfaces. **d** Color-coded charts for qualitative evaluation

The scanning results were compared by using three-dimensional analysis with “shell/shell deviation” software commands regarding the average surface differences with subsequent color-coded charts. Two intraoral scans were registered by using the software’s best-fit algorithm, and overall, three-dimensional comparisons were performed. Since the presence of adjacent soft tissue could increase the range of error, these areas were deleted, along with the gingival margin, to allow superimposition of the clinical crowns. The initial registration involved the selection of three corresponding points on each of the two intraoral scans. Subsequently, automatic fine registration was used to finalize the registration (Fig. 1). The average deviations between the two intraoral scans at all points on the surfaces were computed by using the “shell/shell deviation” function in the program. To remove outlier values, the calculation tolerance value in the program was set at 1.0 mm [23]. In addition, the differences between the two scans were evaluated by means of color histograms.

**Fig. 2:**
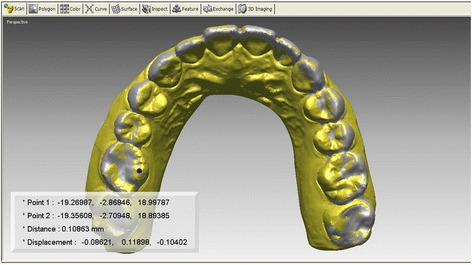
Discrepancies in the three-dimensional direction were calculated in the incisor and molar regions. Three reference points were selected, and the point-to-point distance between each point on the intraoral scan and the corresponding model scan was computed. The point-to-point distance was regarded as the displacement of the intraoral scan to the model scan, and the relative distance was calculated

Deviations in the three-dimensional direction were also calculated in the incisor and molar regions. Three reference points were selected, and the point-to-point distance between each point on the intraoral scan and the corresponding laser-scanned model was computed. As for the reference points, the midpoint between the central incisors and mesiolingual cusp tip of the right and left first molars was determined. The point-to-point distance was regarded as the displacement of the intraoral scan to the laser-scanned model, and the relative distance between each intraoral scan and laser-scanned model was calculated. In addition, the means and standard deviations were computed for each *X*-, *Y*-, and *Z*-coordinate direction to evaluate which direction of the discrepancy contributed to the degree of overall discrepancies (Fig. 2). In order to analyze the differences between the two intraoral scanners, the paired *t* test was used to compare the values using SPSS software package (version 23.0; SPSS Inc., Chicago, IL).

## Results

**Fig. 3:**
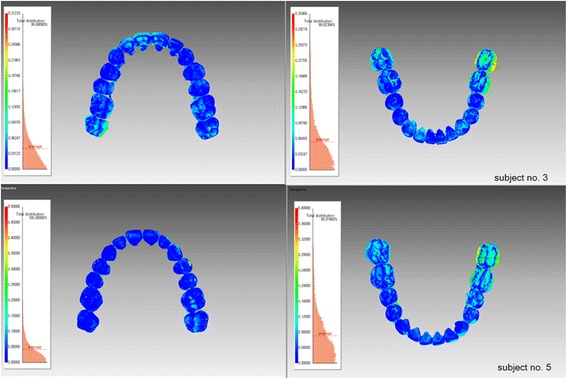
Color-coded charts of the scanning results between the iTero and TRIOS scanners. The two images above are the scans of subject no. 3, and the two images below are the scans of subject no. 5. Discrepancies occurred in the posterior areas, particularly in the mandible on both cases

The average deviations between the two intraoral scans were 0.057 mm in the maxilla and 0.069 mm in the mandible (Table 2). The color histogram showed that local deviations between the two scanners occurred in the posterior area (Fig. 3). In the three-dimensional deviations, the intraoral scans presented a minor displacement to the models; however, there were no statistically significant differences between the two scanners (Table 3).

**Table 2:** Average deviations (mm) between the iTero and TRIOS scanners obtained from a three-dimensional superimposition

	Average deviations	
	Mean	SD
Maxilla	0.057	0.018
Mandible	0.069	0.012

**Table 3:** Three-dimensional discrepancy between each intraoral scan and model in the incisor and molar regions and its comparison between the iTero and TRIOS scanners (unit: mm)

	iTero vs model scan	TRIOS vs model scan	*P* value
	Mean ± SD	Mean ± SD	
Maxilla			
Midpoint between central incisors^*^			
*X*-directional displacement	0.06 ± 0.04	0.04 ± 0.03	0.311
*Y*-directional displacement	0.03 ± 0.10	0.02 ± 0.10	0.758
*Z*-directional displacement	0.01 ± 0.06	0.01 ± 0.09	0.508
First molar mesiolingual cusp, right^†^			
*X*-directional displacement	0.11 ± 0.09	0.08 ± 0.09	0.738
*Y*-directional displacement	− 0.11 ± 0.07	− 0.09 ± 0.08	0.386
*Z*-directional displacement	0.04 ± 0.08	0.02 ± 0.11	0.672
First molar mesiolingual cusp, left^‡^			
*X*-directional displacement	− 0.11 ± 0.11	− 0.06 ± 0.12	0.377
*Y*-directional displacement	− 0.01 ± 0.10	− 0.06 ± 0.07	0.281
*Z*-directional displacement	0.04 ± 0.08	0.05 ± 0.04	0.838
Mandible			
Midpoint between central incisors^§^			
*X*-directional displacement	− 0.02 ± 0.06	0.02 ± 0.08	0.168
*Y*-directional displacement	− 0.07 ± 0.13	− 0.09 ± 0.09	0.757
*Z*-directional displacement	− 0.02 ± 0.09	− 0.09 ± 0.10	0.069
First molar mesiolingual cusp, right^∥^			
*X*-directional displacement	0.01 ± 0.12	0.07 ± 0.10	0.163
*Y*-directional displacement	0.12 ± 0.11	0.12 ± 0.08	0.866
*Z*-directional displacement	0.09 ± 0.17	0.05 ± 0.11	0.410
First molar mesiolingual cusp, left^¶^			
*X*-directional displacement	0.01 ± 0.11	0.02 ± 0.12	0.382
*Y*-directional displacement	0.07 ± 0.11	0.10 ± 0.06	0.539
*Z*-directional displacement	0.15 ± 0.17	0.15 ± 0.07	0.964

*P* values were obtained from paired *t* test. *X*, *Y*, and *Z* directions indicate mediolateral, superoinferior, and anteroposterior directions, respectively.

*SD* standard deviation

^*^In the maxilla, positive value in the *X*, *Y*, and *Z* directions indicates medial, apical, and anterior displacement

^†^In the maxilla, negative value in the *X*, *Y*, and *Z* directions indicates buccal, occlusal, and posterior displacement

^‡^In the maxilla, negative value in the *X*, *Y*, and *Z* directions indicates palatal, occlusal, and posterior displacement

^§^In the mandible, negative value in the *X*, *Y*, and *Z* directions indicates lateral, apical, and lingual displacement

^∥^In the mandible, positive value in the *X*, *Y*, and *Z* directions indicates lingual, occlusal, and anterior displacement

^¶^In the mandible, positive value in the *X*, *Y*, and *Z* directions indicates buccal, occlusal, and anterior displacement

## Discussion

The average deviations between the two intraoral scanners were within 0.07 mm. There is no clear consensus regarding the clinically or medico-legally acceptable amount of error in clinical orthodontics. Many researchers have suggested what they consider to be a clinically significant difference. Hirogaki et al. [24] suggested that orthodontic study models’ accuracy should be about 0.30 mm, while Schirmer and Wiltshire [25] reported that a measurement difference of less than 0.20 mm was clinically acceptable and Bell et al. [26] suggested that a measurement difference within 0.27 mm was clinically insignificant. With regard to the previous studies on the clinical acceptability in plaster models, the differences between two scanners of less than 0.07 mm in our results indicate that both intraoral scanners can be used in clinical orthodontics. Vasudavan et al. [27] compared intraoral scanning and conventional impression techniques for fabrication of orthodontic retainers. The clinical acceptability of retainers did not differ significantly by fabrication method, and the authors concluded that digital scans were considered acceptable [27]. Interestingly, the retainers made from digital scans were preferred significantly more often by the orthodontist than those made from alginate impressions.

In the present study, errors in the plaster model manufacturing process should be taken into consideration. Although alginate impression has potential errors, it is still being used for fabricating a diagnostic model. As plaster model and intraoral scan are used together in the clinics, it is necessary to evaluate the agreement between alginate impression and intraoral scanner.

Confocal laser scanning microscopy is used for intraoral scanner systems [28]. This technique is used to acquire in-focus images from selected depths, a process known as optical sectioning (high-resolution optical images with depth selectivity) [29]. The iTero scanner employs a parallel confocal imaging technique with an array of incident red laser beams [30]. It is important to maintain the scanning wand at a certain focal distance while scanning. The TRIOS intraoral system also works according to the principle of confocal microscopy, with a fast scanning time. A fundamental characteristic of the TRIOS system is the variation of the focal plane without moving the scanner toward the subject being scanned [30]. The TRIOS system has the feature of telecentricity in the space of the subject being scanned, and it is possible to shift the focal plane while keeping telecentricity and magnification ratio [30]. We evaluated the light wave of the iTero and TRIOS scanners using polarization beam splitter and quarter-wave plate (Thorlabs Inc., Newton, NJ). It was found that the light of the TRIOS scanner was polarized, whereas the light of the iTero scanner was not polarized. The TRIOS scanner was found to use linear-to-circular polarization by the quarter-wave plate. A wave plate is an optical device that alters the polarization state of a light wave traveling through it. Two common types of wave plates are the half-wave plate, which shifts the polarization direction of linearly polarized light, and the quarter-wave plate, which converts linearly polarized light into circularly polarized light and vice versa [31]. In consideration of the polarization system of the TRIOS scanner, which might block the scattered reflections, this scanner might have better optical performance than iTero.

In addition, the iTero and TRIOS systems both capture single images of each tooth and produce an assembled virtual model of the whole dentition. This stitching process might produce systematic errors; however, because the stitching algorithms of the iTero and TRIOS scanners are not known, their contribution to such errors cannot be explained. Intraoral conditions such as saliva, breathing, movement of the tongue, and limited oral space can also contribute to scanning inaccuracies. For example, it is difficult for the scanner tip to access the lower posterior areas, owing to tongue movement and limited mouth opening. Flügge et al. [32] found that intraoral scanning was less precise than extraoral model scanning, indicating that the intraoral conditions contribute to the inaccuracy of scans. The accuracy can also be affected by the examiner’s technical skill at intraoral scanning. To avoid such bias, in the present study, intraoral scans were obtained by the same examiner, who had experienced with over 100 cases of intraoral scanning. Long scanning times might induce errors in the stitching process of the captured images; the scanning times tend to decrease as the operator experience increased. In this study, the full-arch scan time for the iTero scanner was an average of 5 min, while the TRIOS scanner was an average of 4 min. However, further studies are needed to assess the scanning accuracy according to the clinician’s experience.

There are few researches about the comparison of iTero and TRIOS scanner for in vivo and full-arch scan. For single abutment, the trueness and precision were higher in TRIOS than iTero scanner [33, 34]. Although Renne et al. [35] reported that trueness and precision for full-arch scanning was higher in iTero than in TRIOS, regarding scanning time, TRIOS was found to have the best balance of speed and accuracy [35]. Although there were no statistically significant differences between the two scanners in this study, some deviations might occur in the posterior areas, particularly in the mandible. In the clinical setting, the use of a careful and extended scanning protocol might improve the scanning results. With the continued development of digital impression technology, we will likely see the elimination of conventional impression taking in the near future.

## Conclusions

Although there were some deviations in visible inspection, there was no statistical significance between the two intraoral scanners.

## Abbreviations

CAD/CAM: Computer-aided design/computer-aided manufacturing
STL: Stereolithography
